# Cost-effectiveness of interventions for children with speech, language and communication needs (SLCN): a review using the Drummond and Jefferson (1996) ‘Referee's Checklist’

**DOI:** 10.1111/j.1460-6984.2011.00084.x

**Published:** 2012-01

**Authors:** James Law, Biao Zeng, Geoff Lindsay, Jennifer Beecham

**Affiliations:** † School of Education, Communication and Language Sciences, Newcastle UniversityNewcastle, UK; ‡ CEDAR, University of WarwickCoventry, UK; § PSSRU, LSE Health and Social Care, London School of Economics and Political ScienceLondon, UK

**Keywords:** children, speech and language difficulties, cost-effectiveness

## Abstract

*Background:* Although economic evaluation has been widely recognized as a key feature of both health services and educational research, for many years there has been a paucity of such studies relevant to services for children with speech, language and communication needs (SLCN), making the application of economic arguments to the development of services difficult.

*Aims:* The study has two aims, namely to review systematically the cost-effectiveness literature related to services for children with SLCN and to highlight key issues that need to be included in future economic effectiveness studies.

*Methods & Procedures:* A comprehensive search of the international literature for the last 30 years was completed and the studies were evaluated against the ‘gold standard’ criteria developed by Drummond and colleagues in 1996 and 2005.

*Outcomes & Results:* Five studies met the review inclusion criteria. All focused on young (2–11 years) children with SLCN and most compared clinic-based and parent-administered interventions. The studies provide variable levels of detail on the key elements needed, but few provided sufficient details of costs to draw comparisons across studies. Only two studies attempted to bring together costs and effectiveness data.

*Conclusions & Implications:* The studies point to the importance of home-based and indirect intervention and, in many cases, emphasize the parental perspective. There is a need for intervention studies to include a cost dimension based on readily comparable methods of establishing unit costs and for greater use to be made of cost-effectiveness analysis more generally.

**What this paper adds**

**What is known on the subject**

There is now an emerging evidence base for the effectiveness of targeted services for children with speech, language and communication needs (SLCN). Interventions for speech and expressive language difficulties look promising, although the picture for other aspects of communication is less clear.

**What this paper adds**

This paper highlights the cost-effectiveness evidence gap and the need to combine effectiveness with cost data. The Drummond and Jefferson criteria for assessing the quality of economic evaluation, well recognized in other fields, are relevant to this area. The five studies highlight the potential merit of indirect work and parental home-based input, but more is needed on the implications of the perspective (education, health, societal, parental) adopted.

## Introduction

The impact of speech, language and communication needs (SLCN) in childhood can be substantial for the individual and to society (Bercow 2008). Although there remains uncertainty as to which children may benefit most from SLCN support in the preschool period, there is evidence which suggests that children who still have a difficulty once they reach primary school are likely to be vulnerable to longer-term consequences. A recent report followed over 11 000 children born in Britain in 1970 through to adulthood. Those with poor vocabulary skills at 5 years were four times more likely to have reading difficulties in adulthood than their peers without SLCN, three times as likely to have mental health difficulties, and twice as likely to be unemployed (Law *et al.* 2009). It would be unsurprising if such a long-term effect did not have significant consequences for society both in terms of the services used and the opportunities lost. This is especially true as the nature of employment has shifted over the last century from manual to white-collar work with huge implications for the salience of communication in the labour market (Ruben 2000).

The costing of public services and especially the field of social welfare has become a critical issue for policy-makers and practitioners alike (Sefton 2000, Beecham 2005). Recently a number of attempts have been made to cost the likely demand on services for those with SLCN together with the resultant benefits. For example, the Audit Commission (2004) reported the cost of services for a child with communication difficulties to the Youth Justice System in England; and a recent report to the Royal College of Speech and Language Therapists modelled not only the cost of services, but also the potential impact of speech and language therapy services for children with specific language impairment and autism, and adults with aphasia and dysphagia (Marsh *et al.* 2010). They estimated that every £1 invested in enhanced speech and language therapy (SLT) for children with specific language impairment had the potential to generate £6.43 through increased lifetime earnings and that the annual net benefit was £623.4 million in England, £36.1 million in Wales, £24.2 million in Northern Ireland, and £58 million in Scotland. Yet beyond such modelling, capturing the financial costs and specific benefits of intervention services to children has proved difficult despite valiant attempts to do so (Hartshorne 2006). Indeed there is nothing comparable with the type of prospective longitudinal analysis available for the High Scope Perry Preschool study which demonstrated that children randomly identified for a specialist preschool intervention had consistently earned more by the time they were 40 years old (Schweinhart *et al.* 2005).

While we have made some progress in understanding the potential effects of SLCN interventions, especially for younger children (Law *et al.* 2003), the economic evaluation of services to children with SLCN is still in its infancy both relative to other predominantly community-based clinical services such as child and adolescent mental health services (Romeo *et al.* 2005, Knapp *et al.* 2008) and in terms of the longer-term costs associated with specific conditions such as childhood antisocial behaviour (Scott *et al.* 2001) and literacy and numeracy difficulties (Gross *et al.* 2009a, 2009b). To address this, Bercow (2008) called not only for a ‘continuum of services designed around the family’, but also for more research into the cost-effectiveness of different interventions and models of collaboration.

### Cost-effectiveness analysis (CEA)

There are three common approaches to economic analysis: cost–benefit analysis, cost–utility analysis and cost-effectiveness analysis (Foster *et al.* 2003). Of these, ‘cost-effectiveness analysis is of most use in situations where a decision-maker, operating with a given budget, is considering a limited range of options within a given field’ (Drummond *et al.* 2005, p. 14). CEA is often the method of choice because it uses the natural unit of measurement in the clinical area concerned and does not rely on a utility measures such as quality-adjusted life year (QALY) nor on complex mechanisms to value resource use and effects on the same metric. CEA is a procedure designed to compare two or more different programmes and identify the additional costs of one service relative to another in the context of the overall effectiveness of the programmes concerned. The ultimate aim of CEA is to calculate the additional cost of producing one extra unit of outcome (Drummond *et al.* 2005). Key to this is the need to be clear about the perspective from which the analysis is made. A societal perspective is the ideal although a public expenditure perspective is becoming more common and is recommended by, for example, the National Institute of Health and Clinical Excellence (NIHCE). Narrower perspectives, such as those incurred by the health sector, or just for the intervention, are likely to miss the impact of SLCN or its treatment on other important cost areas and run the risk of misinforming decision-makers about the true costs

Drummond and Jefferson (1996) stipulated a series of 35 Yes/No questions in what has become known as the ‘Referee's Checklist’ used to judge the quality of a CEA and which has now become a standard way to assess the coverage and quality of a paper, analogous to the PRISMA criteria for the analysis of systematic reviews (Liberati *et al.* 2009) The checklist allows a judgement to be made about whether authors had been explicit about the inclusion of the key ingredients for good-quality cost-effectiveness analyses in three overall categories, namely Study design, Data collection, and Analysis and interpretation. Questions include specific detail about the original study sample; about the type of economic analysis adopted and the rationale for the choice; about the scope of the resource use information reported; and whether unit costs were provided. It also asks about inclusion of key information such as time horizons, discounting and sensitivity analysis.

There is no doubt that while we need to understand what services cost, both to society and to those who use them, it is also important to understand the relationship between costs and outcomes, otherwise we run the risk of asserting that this service is cheaper than another without considering the impact of either service on children. Costs should be clearly linked to outcomes, or health gain, and tap into how much we, as a society, value the outcome in question. As Shonkoff (2004) suggests:

There is an equally compelling imperative about what we might call moral capital. That is to say there are certain things that are important to do because of what they say about our values as a society, above and beyond what they cost in monetary terms. (p. 10)

The present review was carried out following the publication of *The Bercow Report: A Review of Services for Children and Young People (0–19) with Speech, Language and Communication Needs* (Bercow 2008) and the *Better Communication: An Action Plan to Improve Services for Children and Young People with Speech, Language and Communication Needs* (Department for Children Schools and Families (DCSF) 2008) under the auspices of the Better Communication Research Programme commissioned in England in 2009 by the DCSF and awarded to representatives for the universities of Warwick, the West of England (Bristol), London and Newcastle. Indeed, members of the same team provided input to *The Bercow Report itself* (Lindsay *et al.* 2008, 2010). The programme included five projects in its first year, of which economic evaluation is one. These projects are closely linked to one another and it is anticipated that the findings in this study will have a direct bearing on those from the other programmes. The review aimed to identify all cost-effectiveness studies in the field of children's SLCN and to assess them against the quality criteria identified in the Referee's Checklist (Drummond and Jefferson 1996).

## Methods

### Search strategy

Studies were identified by searching electronic databases (Medline, ISI and Scopus. Cochrane and CRD) and scanning relevant reference lists (the last search was completed on 3 March 2010). The following keywords were employed: (1) economic evaluation, cost-effectiveness; (2) speech and language therapy, speech and language delay, speech and language impair* speech and language intervention; and (3) child*. This led to the identification of 1059 studies. All abstracts were examined but only seven were judged to fulfil the relevant criteria, namely those that had to include children with SLCN specifically speech and/or language learning difficulties, had to be written in English and had to include an economic effectiveness analysis. Of those identified two were from the same study (Boyle *et al.* 2007, Dickson *et al.* 2009) and one (Buschmann *et al.* 2009) did not provide sufficient detail about costs. The five included studies were as follows:

- Barnett *et al.* (1988).
- Dickson *et al.* (2009).
- Eiserman *et al.* (1990).
- Gibbard *et al.* (2004).
- Law *et al.* (2006).

### Modifications to the Referee's Checklist

The content of the original Referee's Checklist (Drummond and Jefferson 1996) was reviewed for the purposes of the present study. Questions from the checklist were divided into those that related to prospective intervention studies including empirical data, which were retained, and those that related to economic modelling studies, which were dropped for the purposes of the present review. Items relevant to the present set of studies but for which there were insufficient data were included in the revised checklist. This gave a total of 27 questions in the amended checklist together with grading for the five included studies; these are given in Table 1. The excluded questions are provided in Table 2.

**Table 1:** Included studies against Drummond and Jefferson (1996) criteria with numbering from the original checklist

Code	Item	Barnett *et al.* (1988)	Dickson *et al.* (2009)	Eiserman *et al.* (1990)	Gibbard *et al.* (2004)	Law *et al.* (2006)
1	The research question is stated	N	Y	N	Y	Y
2	The economic importance of the research question is stated	Y	Y	N	Y	Y
3	The viewpoint(s) of the analysis are clearly stated and justified	N	N	Y	Y	Y
4	The rational for choosing the alternative programmes or interventions compared is stated	Y	Y	Y	Y	Y
5	The alternative being compared is clearly described	Y	Y	Y	Y	Y
6	The form of economic evaluation is stated	Y	Y	Y	Y	Y
7	The choice of economic evaluation is justified in relation to the questions addressed	Y	N	N	N	N
8	The source(s) of effectiveness estimated used is stated	Y	Y	Y	Y	Y
9	Details of the design and results of effectiveness study are given	Y	Y	Y	Y	Y
11	The primary outcome measures (s) for the economic evaluation are clearly stated	N	Y	N	N	N
15	The relevance of productivity changes to the study questions is discussed	N	N	N	Y	N
16	Quantities of resources are reported separately from their unit costs	N	N	N	N	N
17	Methods for the estimation of quantities and unit costs are described	N	N	Y	Y	Y
18	Currency and price data are recorded	Y	Y	Y	Y	Y
19	Details of currency of price adjustments for inflation or currency conversion are given	N	N	N	N	N
22	Time horizon of costs and benefits is stated	N	N	N	N	N
25	An explanation is given if costs or benefits are not discounted	N	N	N	N	N
26	Details of statistical tests and confidence intervals are given for stochastic data	N	Y	Y	Y	Y
27	The approach to sensitivity analysis is justified	N	N	N	N	N
28	The choice of variables for sensitivity analysis is justified	N	N	N	N	N
29	The ranges over which the variables are varied are stated	N	N	N	N	N
30	Relevant alternatives are compared	Y	Y	Y	Y	Y
31	Incremental analysis is reported	N	Y	N	Y	N
32	Major outcomes are presented in a disaggregated as well as aggregated form	Y	N	N	Y	N
33	The answer to the study question is given	N	Y	N	Y	Y
34	Conclusions follow from the data reported	Y	Y	Y	Y	Y
35	Conclusions are accompanied by the appropriate caveats	Y	Y	Y	Y	Y

Note: Y, yes; N, no.

**Table 2:** Items excluded from initial Drummond and Jefferson (1996) criteria

Code in Drummond and Jefferson	Item
10	Details of the method of synthesis or meta-analysis of estimates are given (if based on an overview of a number of effectiveness studies)
12	Methods to value health states and other benefits are stated
13	Details of the subjects from whom valuations were obtained are given
14	Productivity changes (if included) are reported separately
20	Details of any model used are given
21	The choice of model used and the key parameters on which it is based on are justified
23	The discount rate(s) is stated
24	The choice of rate(s) is justified

Drummond and Jefferson's checklist makes reference to a ‘doing nothing’ option. In practice in developed countries where relevant health and educational services are available doing nothing is not a realistic or indeed an ethical option. For this reason we have interpreted the ‘doing nothing’ option as meaning doing nothing beyond ‘treatment as usual’ or treatment that the child would have received had the intervention not been in place.

### Reliability of the coding

Cohen's kappa was used to assess the reliability of the Referee's Checklist codings. The independent coding was carried out by the first two authors (James Law and Biao Zeng). A third member of the research team (Jennifer Beecham) moderated the differences. All the coefficients were statistically significant at the *p* < 0.01 level ranging from 0.62 (Dickson *et al.* 2009) to 0.78 (Eiserman *et al.* 1990, Law *et al.* 2006) suggesting that it was feasible to use the Referee's Checklist for this topic.

## Results

Narrative reports are given of the five studies describing their population, number of participants, the interventions, perspective, effectiveness analysis, cost analysis, particular costing considerations, CEA and their conclusions. These reports are followed by a discussion of the five studies’ quality using the checklist. The studies were published between 1988 and 2009 and included a total of 354 children between the ages of 1 year 10 months and 11 years who were seen over a relatively short period of time (fewer than 7 months). Three of the five studies were undertaken in the UK and two in the USA. All studies had two or more arms and all included an economic component. Two of the studies compared two different models of intervention provided by therapists or teaching staff and three included a parent-based intervention. The economic perspectives of the studies varied considerably. The type and severity of speech and language difficulties for which the interventions were provided also varied considerably.

### The studies

#### Barnett et al. (1988)

- *Population:* Children were from middle-income families in the United States qualifying for enrolment in a University Communication Disorders clinic with a mean age of 3;8 years. Twenty-six children had articulation delays, four had language delays and nine had both articulation and language delays.
- *Comparison and perspective:* The authors considered both the dollar costs and the effects of three 13-week intervention periods: (1) home-based intervention, *n* = 10; (2) centre-based intervention, *n* = 10; and (3) both centre- and home-based intervention, *n* = 10. There were nine children in the no intervention arm.
- *Effectiveness analysis*: The study was an RCT. Two standardized child language tests were used to assess effectiveness: the Preschool Language Scale and the Arizona Articulation Proficiency Scale.
- *Cost analysis*: The report covers intervention costs to the health sector and the costs associated with parent time.
- *Particular cost considerations:* (1) the clinic paid a fixed overhead rate to the university in which it was housed; (2) the measurement of time use; and (3) students and parents were paid nothing so were valued at zero and at average/minimum wage levels respectively.
- *Cost-effectiveness analysis:* This study compared costs of per child for the three different interventions (home-based, centre-based and combined), but did not undertake a CEA, nor were sensitivity analyses reported.
- *Conclusion:* The study concluded that the home-based intervention was more efficient because the centre-based intervention was both more expensive and less effective. The study was limited in size and duration, but the authors suggest that parent-delivered service should be investigated further.

#### Dickson et al. (2009)

- *Population:* Children with ‘primary language impairments’ aged between 6 and 11 years in the UK.
- *Comparison and perspective*: The study examined both costs and effects of five interventions: direct-individual therapy, *n* = 34; direct-group therapy, *n* = 28; indirect-individual therapy, *n* = 33; indirect-group therapy, *n* = 29; and standard therapy, *n* = 28. Each programme ran for 15 weeks. Only partial intervention costs were estimated.
- *Effectiveness analysis:* Effectiveness was based on data from an RCT comparing the pre- and post-test scores assessed using the Clinical Evaluation of Language Fundamentals (CELF-III UK).
- *Cost analysis:* The study took into account the salary and travel costs associated with the intervention, excluding costs for support staff (e.g. administration), materials, venue and overheads.
- *Particular cost considerations:* Difficulties in estimating the time spent on the intervention were discussed, for example accounting for time scheduled for direct therapy that could not always be productively reallocated if a child was absent at short notice, and that non-attendance by a child in individual therapy represented a real time cost to the therapist or assistant. The costs included non-contact time associated with preparation for the therapy sessions and time taken in travelling.
- *Cost-effectiveness analysis:* The mean costs per child and per therapy mode, were calculated for each arm of the study.
- *Conclusion:* The analysis showed that there was no significant difference of effectiveness across these four modes. The indirect therapy, particularly indirect group therapy, was the least costly of the intervention modes while direct individual therapy was the most costly option. The implications for implementation in practice were discussed.

#### Eiserman et al. (1990)

- *Population:* Three- to five-year-old children with ‘moderate speech disorders’ from middle class backgrounds in the United States.
- *Comparison and perspective:* The authors considered both the costs and the effects of two intervention programmes, one home-based intervention (*n* = 20) and the other on a clinic-based intervention (*n* = 20). Each programme lasted 7 months. Intervention costs were calculated.
- *Effectiveness analysis:* Measures of effectiveness included language, speech and naturalistic utterance analysis, and both pre- and post-test results within and between both programmes were compared. There was no significant difference between the home- and the clinic-based interventions. The speech and language assessments and analysis used in this study included the Goldman–Fristoe Test of Articulation, the Preschool Language Scale (PLS), the Patterned Elicitation Syntax Test (PEST), which was only used at pretesting, the Test for Auditory Comprehension of Language (TACL-R) and Natural Language Sample; the latter two were administered only at post-testing.
- *Cost analysis:* The calculation of intervention costs covered staff facilities, transportation, therapy materials and parent time.
- *Particular costing considerations:* Parent time was assigned as a monetary value of US$9/h based on median women's weekly earnings. The estimation of parent cost in the home-based programme was based on: (1) programme records of the actual time parents spent during the home visit; (2) the amount of time spent in the parent support group; and (3) the amount of time the programme recommended for the intervention between sessions. In the clinic-based programme, the estimation of parent cost included the time spent in the parent support group and the time spent in transporting children to the clinic.
- *Cost-effectiveness analysis:* Although the study was described as a CEA, the analysis only compared the intervention costs given that there was no significant difference between the home- and the clinic-based interventions. No sensitivity analysis was performed.
- *Conclusion:* The study concluded that the trained mothers could provide therapy to their moderately speech-disordered children as effectively as professional speech therapists. However, the findings suggest a possible drawback to the ‘parent-as-therapist’ approach as the children and mothers in the home-based intervention were less likely to engage in free, spontaneous communication with each other. The home-based programme cost over 20% more than the clinic-based programme when the value of parent time was included.

#### Gibbard et al. (2004)

- *Population:* Children with expressive language delays in the UK with a mean age of 2 years 3 months.
- *Comparison and perspective*: The study examined both cost and effects of two programmes: parent-based intervention (PBI) (*n* = 12) and routine practice (*n* = 10), for preschool children with expressive language delay. Each programme lasted 6 months. The authors raised the issue of perspective and discussed the parents’ cost but only ‘direct therapy cost’ was included in the analysis. They conceded that ‘the perspective of the current study was narrow’.
- *Effectiveness analysis:* The effectiveness data came from a comparison between PBI and routine practice. Effectiveness was measured using the Reynell Developmental Language Scales (RDLS), the Preschool Language Scales (PLS-UK) and mean length of utterance (MLU).
- *Cost analysis:* The study considered intervention costs and parents’ out-of-pocket travel costs and also recorded parent time. No other sector costs were taken into account. The sources of all values were clearly identified.
- *Cost-effectiveness analysis:* The cost per child was compared for each programme from the care provider's perspective. Furthermore, the point estimate of the cost per additional unit of outcome generated by PBI for each of the six measures employed in the study was calculated using an incremental analysis.
- *Conclusion:* PBI was found to improve children's language more than routine practice. In a simple comparison of direct treatment costs PBI appears the more costly intervention. The increased outcomes generated by PBI meant that for all measures the cost per outcome (outcome/cost) was lower for PBI than for usual practice. However, the incremental CEA (the additional costs imposed by PBI over usual practice compared with the additional benefits it delivers; Gibbard *et al.* 2004: 240) showed that the cost to the Trust of obtaining the improved outcomes on the PLS (expressive language scale) was £3.82 (1999 prices). The report includes a useful discussion of the impact of small sample sizes on findings from cost analyses.

#### Law et al. (2006)

- Population: Children in the UK with pronounced speech and language delays secondary to general learning disabilities from socially disadvantaged backgrounds with a mean age of 2 years 9 months.
- *Comparison and perspective:* The study examined both costs and effects of two types of provision: (1) two Early Years Centres (EYC A and B, *n* = 58) specializing in provision for children with delayed speech and language development; and (2) routine speech and language therapy offered by the UK National Health Service (NHS), *n* = 33. EYC A programme lasted 10 weeks and EYC B lasted 6 weeks. The duration of routine speech and language therapy was not reported. The authors adopted a societal perspective although parental costs were omitted.
- *Effectiveness analysis:* The study compared the intervention results for the EYC and NHS groups. Effectiveness was measured using the Preschool Language Scale and the British Ability Scales.
- *Cost analysis:* Costs for the interventions were included, but parent time and out-of-pocket costs, other sector costs or productive losses were not included in analysis.
- The intervention costs included staff time, administration and facilities, equipment, and materials/supplies.
- *Particular cost considerations:* The EYC intervention had a number of functions. It provided speech and language therapy and an educational curriculum but also acted as a substitute for nursery care and provided other recreation opportunities for the children. The impact on the costs of nursery care was explored. The interventions were provided in an inner city area.
- *Cost-effectiveness analysis:* This analysis compared the costs of the two types of provision. EYC was slightly more expensive than NHS SLT plus nursery care, but not significantly so, although this finding may be due to the relatively small sample size. No incremental analysis of costs and consequences of alternatives was undertaken.
- *Conclusion:* The conclusions are based on the cost per child and set in the context of the effects of each programme and the findings from other studies that have investigated a similar question. The researchers maintained that the cost of speech and language intervention was relatively low compared other health services.

Turning to the application of the set Drummond *et al.*'s criteria adopted in this review it is clear there was considerable variability in terms of the reporting across the five studies (Table 1). Only one paper included what Drummond and colleagues call a ‘doing nothing’ alternative intervention. Four of the five studies employed random allocation to groups. No modelling studies were included although one had since been completed (Marsh *et al.* 2010) and data in all the included studies were collected prospectively. Although the methods for cost estimation were usually presented the perspective adopted was commonly very narrow and only condition specific outcome measures were used. Two studies used incremental CEA (Gibbard *et al.* 2004, Dickson *et al.* 2009). A common theme was that there was relatively little recognition of the likely uncertainty around the cost findings. Even with the reduced version of the checklist, seven of the 27 questions were not addressed by any of the studies. Eight questions obtained affirmative responses for all five studies. If we look at the overall coding of individual studies we see that the range of affirmative scores went from 12 for Barnett *et al.* (1988) to 18 for Gibbard *et al.* (2004).

A key feature of the three studies that evaluated parental training is their estimation of family-borne costs for travel and/or time (Barnett *et al.* 1988, Eiserman *et al.* 1990, Gibbard *et al.* 2004). Parents obviously have an important role in home-based treatment and two studies (Barnett *et al.* 1988, Eiserman *et al.* 1990) show how the costs increase considerably if parental time is included. Barnett *et al.* (1988) found that the home-based intervention was less expensive than the clinic-based intervention even adding in the cost of parents. There have been differences in the way that parental costs have been calculated, leading to different interpretations of the data. For example, Eiserman *et al.* (1990) found that their home-based programme cost over 20% more than the clinic-based programme; while Gibbard *et al.* (2004) show that parents’ travel time to attend sessions can be considerable and that to attend the PBI sessions parents might have incurred additional out-of-pocket expenses for childcare. Three of the five studies suggest that a family- or parent-based therapy is effective for SLCN or at least no less effective than traditional SLT service, a conclusion which is broadly aligned to the Cochrane review of intervention studies in this field (Law *et al.* 2003).

The Drummond and Jefferson checklist has enabled a useful insight to be made into the state of the art of economic analysis in the field of SLCN. While there are some components of CEA the studies cover quite well, others are not addressed. This is, of course, partly a historical matter. Some of these studies predate the recent developments in techniques for health economics and it also reflects an historically lower interest on the part of researchers and research funders in including an economic component in evaluations of SLCN interventions. In short, the analysis demonstrates that there are many gaps in these studies when they are examined against these criteria; gaps which should be addressed in future studies.

## Discussion

The review has uncovered a small group of cost-effectiveness papers published over a 21-year period. There is evidence for the effectiveness of SLCN interventions, further supporting the findings of Law *et al.* (2003) and suggesting that the first threshold for undertaking economic evaluation has been met. If there were no such evidence of effectiveness, there would be little value in developing cost-effectiveness studies. The studies demonstrate both that child outcomes may be improved, when parents are actively involved in the intervention relative to when they are not, and that these interventions are less costly if the costs attributable to parents are excluded. At face value this is rather obvious. If you cost more aspects of an intervention they will almost invariably be higher. But this can be more complex with parental intervention and it depends on whether the resultant parenting activity is in addition to what might be considered their normal parenting or whether it replaces what they do with new skills. Therefore asking parents to spend time actively training their child for 1 h everyday is substantively different from asking them to change their parenting style whereby they are generally more responsive and correspondingly less didactic in their interaction style. This highlights the need to provide appropriate level of costs, distinguishing between a service provider and a societal perspective to the costing. A societal perspective provides the most comprehensive option but may not be of specific relevance, say, to educational providers. In the case of children with SLCN the costs covered by health and education services need to be supplemented by parental costs. Similarly given the compound needs of many of these children the broad range of health costs such as visits to other professionals (paediatrics, audiology, psychology etc.) should be included. We also have some evidence for the relative costs of group compared with individual interventions. Although the age range of the children in the studies goes through to 11 years, most of them focused on much younger children. We can be reasonably confident that there are no other studies beyond those identified here that would have met our inclusion criteria for SLCN intervention and cost-effectiveness. There may be evaluations of early education for socially disadvantaged children or for those with reading difficulties that may have assessed language outcomes. There are some other studies that collected information on the resources used to deliver the intervention being evaluated. For example, we identified one comparing the effectiveness of the Hanen Parent Programme (HPP) with conventional clinical therapy in which the authors identified the number of staff hours taken to provide each intervention (Baxendale and Hesketh 2003). Such studies represent an important ‘transition’ towards economic evaluation; the next steps for such studies are the estimation of the costs of staff time and other resources used and the explicit linkage of the resulting total costs to outcomes.

There are a number of general observations that can be made about the process adopted and the studies identified. The checklist has proved to be helpful because it exposes CEAs in the SLCN field to the type of criteria widely adopted in other areas of healthcare delivery. Some of the intervention studies discussed here predate the introduction of the Drummond criteria and for at least one study the research team did not appear to include an economist. Such omissions would be relatively easy to address in future analyses and to do so would make a considerable difference to our understanding of how best to use SLCN resources. It is also important to note that our approach to identifying studies meant that they were only included if there was a comparison group. Both effectiveness and cost-effectiveness studies require such a method for these are relative concepts; is this intervention more (cost-) effective than another? However, this has meant that analyses of national or routinely collected data have not been reviewed and yet they often report models and findings that are relevant to both policy and practice (for example, Allen 2011).

If we then turn to the details of the studies there is a specific issue related to the study sample sizes and thus the confidence attached to the results. All the studies had relatively small samples. Only one included a formal sample size calculation and none estimated the sample size using a cost or cost-effectiveness measure. The sample size needed to generate sufficient statistical power to detect a difference between an intervention and a comparison group is likely to be much lower for an outcome study than it is for a CEA (Drummond and Davies 1991). The second issue is that although the studies all calculate costs they do so inconsistently, making direct comparison across studies difficult. Gold *et al.* (1996) propose the use of a reference case, a preferred set of reporting standards, and recommend comparing incremental cost-effectiveness ratios. More recently, cost-effectiveness acceptability curves have been recommended as a way of better presenting these data (Fenwick *et al.* 2001, Fenwick and Byford 2005).

It is clear from the popular response to *The Bercow Report* and indeed the countless reports of the critical role that communication plays in our highly sophisticated literate society that a child with SLCN represents a real challenge to parents and teachers alike. Interventions of proven efficacy are needed to enhance a child's children's capacity to engage with school, family and friends, but also to improve their access to employment and well-being as adults. Indeed it could be argued that it is enhancing the child's school readiness and thus their ability to participate in society that should be the principal objective of any speech and language intervention.

Ultimately we would want a series of comparable analyses addressing specific questions that would allow direct comparisons across studies and ultimately meta-analysis. Drummond's Referee's Checklist goes some way to addressing this issue by highlighting the types of information that all such studies should include. Clearly the first step is good, comprehensive data on all the services that children receive. This is particularly important when it comes to services that could be offered through a number of routes. SLCN interventions can be offered at nurseries and schools both mainstream and specialist, through parent-led training programmes, through direct or indirect speech and language therapy, and even through computer- or internet-based programmes. Although the adoption of a single perspective, such as recording input from the intervention provider, is common, it is rarely sufficient to identify the true cost. As these studies show, if SLCN interventions include parents in an active role providing instruction to the child on a regular basis outside the context of the therapy received from the specialist, parental input costs should be costed. If researchers do not include the cost of that parental input, whether functional costs (transport and the like) or the costs of time spent reinforcing the aims of the intervention, the overall cost of the programme is likely to be misrepresented. It is also possible that minimizing the cost of one service simply shifts the costs of SLCN onto another agency. For example, a cheaper and less effective intervention provided by the health sector may mean that the education services must pick up additional costs of supporting pupils with SLCN. While this is clearly true of targeted intervention studies of the type described in the present review it is especially important to take this into consideration as the number of universal or preventative studies increases.

## Conclusions

The study of the economic effectiveness of intervention studies for children with SLCN is in its infancy. The published literature indicates that positive outcomes are achievable and point to cost-effective models of service delivery, taking into account the specific nature of the activity carried out by the parent. SLCN interventions, like most services delivered through the public sector, will be competing for limited resources not only just with a range of interventions for other conditions, but also between interventions for SLCN. If effective commissioning decisions are to be made, more and better quality economic evaluations are needed to inform this process.
